# Direct cloning and heterologous expression of the salinomycin biosynthetic gene cluster from *Streptomyces albus* DSM41398 in *Streptomyces coelicolor* A3(2)

**DOI:** 10.1038/srep15081

**Published:** 2015-10-13

**Authors:** Jia Yin, Michael Hoffmann, Xiaoying Bian, Qiang Tu, Fu Yan, Liqiu Xia, Xuezhi Ding, A. Francis Stewart, Rolf Müller, Jun Fu, Youming Zhang

**Affiliations:** 1 Shandong University–Helmholtz Institute of Biotechnology, State Key Laboratory of Microbial Technology, School of Life Science, Shandong University, Shanda Nanlu 27, Jinan, 250100, People’s Republic of China; 2 Department of Genomics, Dresden University of Technology, BioInnovations-Zentrum, Tatzberg 47-51, Dresden, 01307, Germany; 3 Helmholtz Institute for Pharmaceutical Research, Helmholtz Centre for Infection Research and Department of Pharmaceutical Biotechnology, Saarland University, PO Box 151150, Saarbrücken, 66041, Germany; 4 Hunan Provincial Key Laboratory for Microbial Molecular Biology-State Key Laboratory Breeding Base of Microbial Molecular Biology, College of Life Science, Hunan Normal University, Changsha, 410081, People’s Republic of China

## Abstract

Linear plus linear homologous recombination-mediated recombineering (LLHR) is ideal for obtaining natural product biosynthetic gene clusters from pre-digested bacterial genomic DNA in one or two steps of recombineering. The natural product salinomycin has a potent and selective activity against cancer stem cells and is therefore a potential anti-cancer drug. Herein, we separately isolated three fragments of the salinomycin gene cluster (*sal*O*-orf*18) from *Streptomyces albus* (*S. albus*) DSM41398 using LLHR and assembled them into intact gene cluster (106 kb) by Red/ET and expressed it in the heterologous host *Streptomyces coelicolor* (*S. coelicolor*) A3(2). We are the first to report a large genomic region from a Gram-positive strain has been cloned using LLHR. The successful reconstitution and heterologous expression of the salinomycin gene cluster offer an attractive system for studying the function of the individual genes and identifying novel and potential analogues of complex natural products in the recipient strain.

Red/ET recombineering in *E. coli*^1^,^2^, is a powerful technique for the genetic engineering of natural product biosynthetic pathways, especially for large polyketide synthetase (PKS) as well as nonribosomal peptide-synthetase (NRPS)^3^,^4^,^5^,^6^. Recently, this technique was used to clone large biosynthetic gene clusters from a complex DNA source into a vector by linear plus linear homologous recombination (LLHR)^7^. LLHR is mediated by the full-length Rac prophage protein RecE, an exonuclease, its partner RecT, a single-strand DNA-binding protein, and Redγ, an inhibitor of the major exonuclease. RecA, a repair protein, is also included^8^. Fu *et al.*, 2012 cloned ten hidden biosynthetic pathways from digested genomic DNA of Gram-negative *P. luminescens* using LLHR, and two of these have been successfully expressed in *E. coli*^7^,^9^. Many gene clusters have also been cloned by this method, including the syringolin, glidobactin, and colibactin gene clusters^10^,^11^,^12^, and all are from Gram-negative strains.

An emerging idea in cancer biology is that tumors harbor a group of cells, known as cancer stem cells (CSCs), which have the unique ability to regenerate cancers^13^,^14^. In addition to promoting tumor growth, growing evidence indicates that CSCs may be responsible for cancer recurrence, resistance to conventional treatments and metastasis^15^,^16^,^17^,^18^. Recently, Lander *et al.*, 2009 showed that salinomycin can selectively kill breast CSCs after screening 16,000 compounds^19^. Further studies revealed that salinomycin has potent and selective activity against other cancer cell lines^20^,^21^. *In vitro* data revealed that salinomycin pre-treatment reduced the tumor-seeding ability of cancer cell lines greater than 100-fold over the chemotherapy drug paclitaxel. Furhtermore, salinomycin reduced mammary tumor size in mice to a greater extent than paclitaxel^19^.

Salinomycin is produced by *Streptomyces albus*^22^ and has been used to prevent *Coccidioidomycosis* in poultry and alter gut flora to improve nutrient absorption in ruminants^23^. The compound interferes with potassium transport across mitochondrial membranes, thus reducing intracellular energy production. It may also disrupt Na^+^/Ca^2+^ exchange in skeletal and, in some cases, cardiac muscle, allowing a fatal accumulation of intracellular calcium^24^.

Earlier results revealed that the polyketide chain of salinomycin is synthesized by an assembly line of nine PKS multienzymes (*sal*AI–IX). The nine PKS genes are collinearly arranged in the cluster. Four of these multienzymes (*sal*AIV, *sal*AVI, *sal*AVII, and *sal*AIX) each catalyze a single extension module, while the other five (*sal*AI, *sal*AII, *sal*AIII, *sal*AV, and *sal*AVIII) encode two extension modules. In addition to the nine PKS genes, some other genes play vital roles in salinomycin biosynthesis^25^,^26^. Upstream of the PKS genes, the adjacent *orf*1, *orf*2, and *orf*3 do not belong to the salinomycin cluster, but *sal*N and *sal*O encode putative regulatory proteins. SalP and SalQ are involved in the formation of the butyrate extender unit for salinomycin biosynthesis, and inactivation of *sal*P and *sal*Q reduced the yields of salinomycin by 10% and 36%, respectively when compared to wild-type^26^. Downstream of the PKS genes, *orf*18 is predicted to encode a peptidyl carrier protein, and targeted inactivation of *orf*18 results in a 50–60% reduction in salinomycin production compared to wild-type^25^.

Herein, we report the cloning of the 106-kb salinomycin gene cluster (*sal*O*-orf*18) from the genomic DNA of *Streptomyces albus* DSM41398 by three rounds of direct cloning followed by assembling. All of the genes are oriented in the same direction and under the original promoters. The gene cluster was introduced into *S. coelicolor* A3(2) for successful heterologous production of salinomycin.

## Results

### Constructing a BAC vector for direct cloning of the salinomycin gene cluster by quadruple recombineering

**Figure 1:**
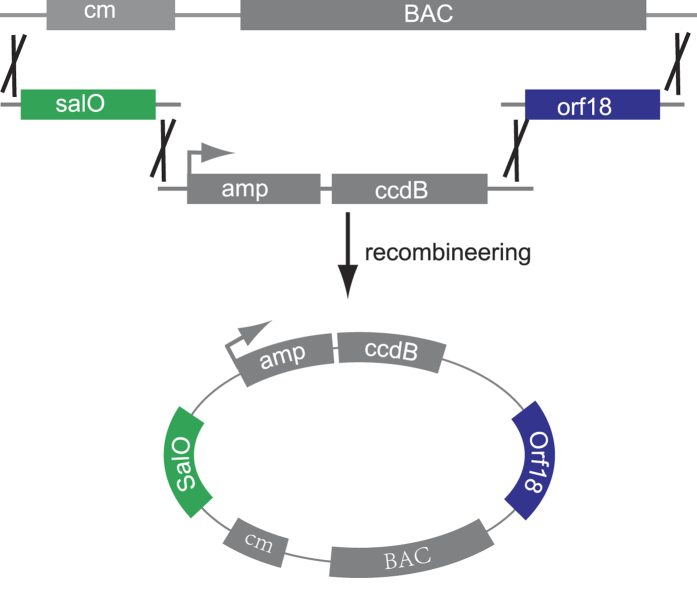
Quadruple recombineering of the BAC vector for direct cloning of the salinomycin gene cluster. pBeloBAC11 was linearized by *Bam*HI, and three fragments (*sal*O, *amp*-*ccd*B, and *orf*18) were obtained by PCR. An ampicillin resistance gene and *ccd*B were co-expression under the same promoter.

In order to construct a vector for direct cloning of the salinomycin gene cluster, the four fragments (backbone of pBeloBAC11, amp-ccdB, *sal*O, and *orf*18) each had a 50-bp overlapping sequence, as illustrated in Fig. 1, and were co-electroporated into GB05dir-*gyrA*_*462*_^5^, a CcdB-resistant *E. coli* strain containing the mutation GyrA R462M^27^,^28^ and LLHR-proficient recombinase (RecET, Redγ, and RecA), to form the BAC vector by quadruple recombineering.

The BAC vector contained a homology arm to *sal*O (292 bp) and *orf*18 (238 bp) and a cassette of the counterselection marker CcdB, which can be used to delete the background from the original BAC vector for direct cloning. A CcdB function test was performed as described previously^5^.

### Direct cloning of the salinomycin gene cluster

As mentioned above, *sal*O encodes putative regulatory protein and *orf*18 is an essential factor for salinomycin production. Additionally, the restriction site (*EcoR*V), which can be utilized for direct cloning, is located in *sal*O and *orf*18. Thus, we attempted to directly clone the 106-kb fragment (*sal*O-*orf*18) using one and two-step recombination reactions^7^ with the BAC vector but were unsuccessful.

**Figure 2:**
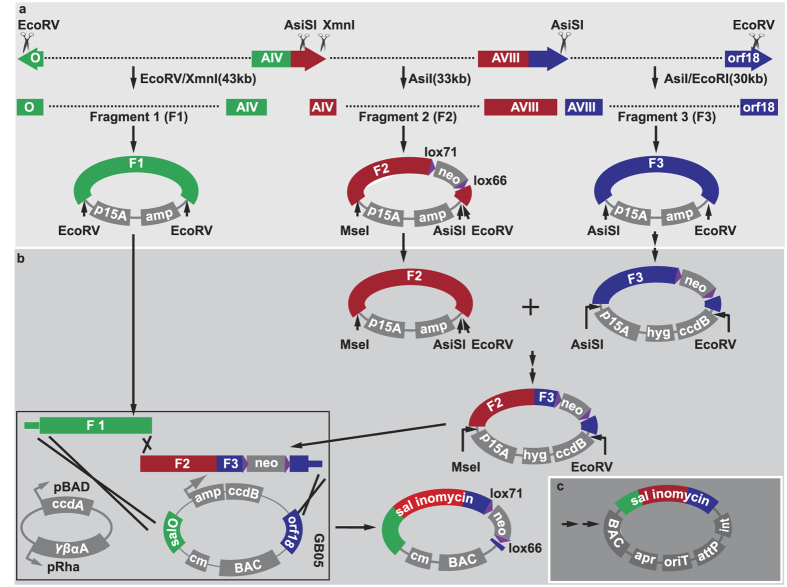
Diagram of direct cloning and assembling of the salinomycin gene cluster and engineering for conjugation and integration. (**a**) Genomic DNA was digested by restriction enzymes to produced three fragments, which were recombined with p15A-amp after direct cloning. Fragment F2 was isolated using the neomycin selection marker. (**b**) Three fragments were assembled. Fragments F2 and F3 were assembled by a ligation reaction. F1 and F2&3 were assembled together by triple recombineering to produce pBeloBAC11-sal-lox71-neo-lox66. (**c**) The neomycin selection marker was deleted by Cre from the pBeloBAC11-sal-lox71-neo-lox66 plasmid, and the integrase-attP-oriT-apramycin cassette was inserted into the noncoding sequence to generate the final construct, pBeloBAC-sal-int-attP-oriT-apr. hyg, hygromycin resistance gene; amp, ampicillin resistance gene; neo, neomycin resistance gene.

Hence, we divided the gene cluster into three fragments for direct cloning (Fig. 2a). We directly cloned the fragments of *sal*O*-sal*AIV (F1) and *sal*AIX*-orf18* (F3) using one step of LLHR^7^ with an efficiency of 4/24 and 1/24, respectively (Fig. S1a,c). We directly cloned the fragment of *sal*AIV*-sal*AVIII (F2) by a two-step recombination with an efficiency of 8/24 (Fig. S1b). Due to the repeated sequence in *sal*AIV*-sal*AVIII (Fig. S2), we were unable to directly clone this fragment by one step of LLHR. Therefore, this fragment was isolated using a neomycin selection marker flanked by lox71-lox66, which could be utilized to delete the selection marker conveniently in the assembling procedure. The three desired fragments were inserted in plasmids p15A-amp-F1, p15A-amp-F2-lox71-neo-lox66, and p15A-amp-F3, respectively.

### Assembling of the salinomycin gene cluster and engineering for conjugation and integration

Figure 2b show the assembling procedure to reconstitute the entire cluster. F2 and F3 were ligated using the original restriction site of *AsiS*I/*EcoR*V in the gene cluster, which did not cause any open reading frame shift. The neomycin selection marker was deleted by Cre from the plasmid p15A-amp-F2-lox71-neo-lox66 to produce p15A-amp-F2. Modifications were made to p15A-amp-F3 with two steps of recombineering. The neomycin selection marker flanked by lox71-lox66 was inserted into the non-coding sequence of F3 in the first recombineering step. The second recombineering step replaced the ampicillin selection marker with the *hyg-ccd*B cassette to produce p15A-hyg-ccdB-F3-lox71-neo-lox66. F3 was excised by *AsiS*I/*EcoR*V and inserted into the *AsiS*I/*EcoR*V site in p15A-amp-F2 by ligation to produce p15A-amp-F2&3-lox71-neo-lox66.

The ampicillin selection marker of the previous ligation product was replaced by the hyg-ccdB cassette to produce the plasmid p15A-hyg-ccdB-F2&3-lox71-neo-lox66. The plasmid p15A-amp-F1 was digested by *EcoR*V to release the fragment F1, and p15A-hyg-ccdB-F2&3-lox71-neo-lox66 was digested by *EcoR*V/*Mse*I to excise F2&3-lox71-neo-lox66. The two fragments overlapped by 592 bp, and each fragment had a homologous arm with previously constructed BAC vector. The BAC vector was transformed into GB05 cells harboring the plasmid pSC101-ccdA-gbaA. As CcdB is toxic, we induced CcdA, that inactivates the CcdB toxin, by rhamnose in the liquid medium or culture plates. The two previous linearize fragments were co-transformed into GB05 cells containing the BAC vector and the expression plasmid (pSC101-ccdA-gbaA) to produce pBeloBAC11-sal-lox71-neo-lox66. We verified pBeloBAC11-sal-lox71-neo-lox66 using three restriction enzymes, the results (Fig. S3) showed that the pBeloBAC11-sal-lox71-neo-lox66 was correct.

To introduce the gene cluster into a heterologous expression host, few necessary elements were engineered before conjugation. The two step engineering procedure for conjugation and integration is diagrammed in Fig. 2c. Finally, the gene cluster was introduced into *S. coelicolor* A3(2) by conjugation and integrated into its chromosome.

### Heterologous production of salinomycin in *S. coelicolor* A3(2)

The genetic organization and promoters of the obtained salinomycin gene cluster are identical to those of the original producer *S. albus* DSM41398. After conjugation, the exconjugant colonies were confirmed by PCR and subsequently analyzed for heterologous salinomycin production. The salinomycin gene cluster was successfully inserted into the attB site of *S. coelicolor* A3(2) (Fig. S4).

**Figure 3:**
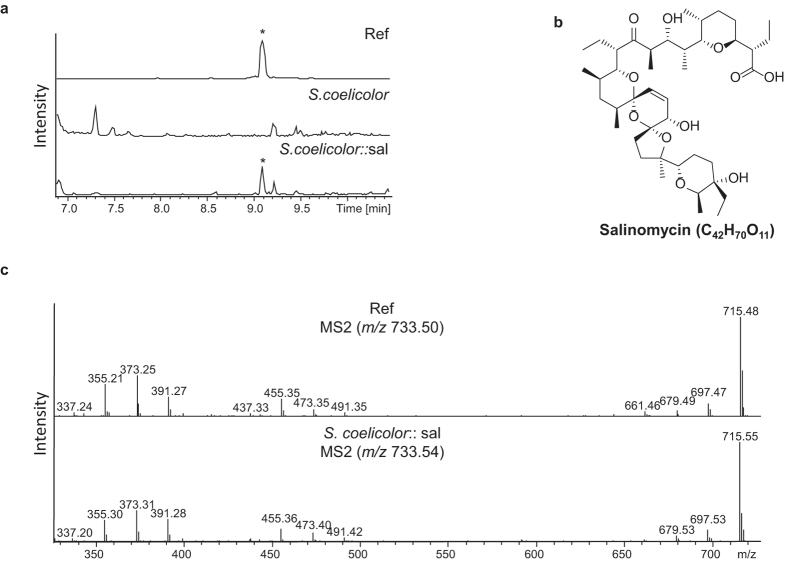
Heterologous salinomycin production. (**a**) HPLC-MS analysis (base peak chromatograms (BPC) *m/z* 200–2000+ All MS) of the salinomycin standard (Ref), the wild-type *S. coelicolor* A3(2) and mutant *S. coelicolor::sal*. Salinomycin is indicated by an asterisk. (**b**) MS^2^ fragmentation patterns of precursor *m/z* 733.5 [M–H_2_O+H]^+^ in standard salinomycin and in *S. coelicolor::sal* mutant.

The metabolite profiles of the wild-type *S. coelicolor* and the mutant strains *S. coelicolor::sal* were analyzed by HPLC-MS and compared with the salinomycin standard (Fig. 3a (Ref)). Thus, we were able to identify Salinomycin in extracts of the mutant strains *S. coelicolor::sal* via HPLC-MS (Fig. 3a,b) and heterologous expression could be unambiguously confirmed by comparing MS^2^ fragmentation pattern (Fig. 3c).

## Discussion

Over the past several decades, numerous multifunctional megasynthases have been identified, cloned, sequenced, engineered, and heterologously expressed in suitable hosts. Traditionally, natural product biosynthetic gene clusters were retrieved from a single cosmid or reconstructed from several cosmids within a genomic library of the natural producer stain, which was time consuming due to subsequent cloning steps following the screening process from a genomic library^4^,^29^.

LLHR-mediated recombineering was ideal for direct cloning of the salinomycin gene cluster from pre-digested genomic DNA after one or two steps of recombineering^7^. Red/ET recombineering has traditionally been applied for heterologous expression of biosynthetic pathways to modify the biosynthetic pathways^30^.

The failure to directly clone the 106-kb fragment with the BAC vector may have resulted from several considerations. First, the recombineering efficiency is very low for large fragments. Although the developed method of direct cloning is efficient for cloning up to ~52-kb fragments from a bacterial genome^7^, it is limited by inefficient co-transformation of two linear molecules, especially for long fragments (106 kb). Moreover, the gene cluster contains GC-rich sequences. We studied the impact of the GC content on the recombineering efficiency and found that it was decreased for sequences with high GC content (data not shown). Second, enrichment of the target DNA is difficult after extracting the genomic DNA. Genomic DNA is susceptible to shearing forces associated with mechanical destruction and degradation by nuclease activity. Therefore, it is difficult to obtain the intact salinomycin biosynthesis gene cluster, especially for *S. albus* DSM 41398, the gram-positive strain. Third, previous data revealed that the Redβ monomer anneals ~11 bp of DNA, and the smallest stable annealing intermediate requires only 20 bp of DNA and two Redβ monomers^31^. In this study, we found that most of the colonies resulted from self-circularization of the vector used for direct cloning after recombineering although there were no obvious homologous regions in the backbone of the vector. As a result, it is difficult to screen the correct clone from thousands of self-circularized vectors.

In parallel to our LLHR-mediated direct cloning, the other DNA cloning methods for bioprospecting have their distinct merits. LLHR-mediated RecET direct cloning was not accessible to metagenomic DNA. Bioprospecting of metagenomics needs DNA synthesis and assembly method. *Streptomyces* phage ϕBT1 integrase-mediated *in vitro* site-specific recombination could assembly the 56 kb epothilone biosynthetic gene cluster using modules as units. The authors didn’t prove that the complete gene cluster with *att* site scars could be expressed in a heterologous host^32^. The incorrect linker between modules might affect the biosynthesis^33^. An intact DNA sequence can be obtained by the Gibson assembly^34^,^35^, which is the most efficient ‘chew back and anneal’ method^36^,^37^,^38^. The Gibson assembly was also proved to be capable of direct cloning of a 41 kb conglobatin biosynthetic gene cluster^39^. Much larger DNA fragment can be directly cloned by transformation-associated recombination (TAR) in yeast *Saccharomyces cerevisiae*^40^,^41^. However unregulated yeast homologous recombinase might cause rearrangement of repetitive PKS/NRPS biosynthetic DNA sequences. The *ori*T-directed cloning for Gram-negative bacteria relies on available genetic tools to insert conjugation elements on the genome by two elaborated vectors. Although it is not straight forward, it has a capacity of cloning regions up to 140 kb from the genome of *Burkholderia pseudomallei*^42^. The phage ϕBT1 integrase-mediated direct cloning was developed for Gram-positive bacteria *Streptomyces*. It has the similar logic to *ori*T-directed cloning, which starts with integration of a capture vector by genome engineering at two spots, but both excision and circularization happen in the original bacteria^43^. If *Bacillus subtilis* is justified as a suitable heterologous host for a biosynthetic gene cluster, its genome can be used as a vector for direct cloning of giant DNA, which has the potential to overcome the capacity limit of the BAC vector^44^.

Compare to above methods our LLHR-mediated direct cloning has a significant feature. It is a genetic tool in *E coli*, which is simple, convenient and cost-effective. The important improvement in this study is to combine RecET mediated direct cloning and *lambda* Red mediated plasmids stitching to hierarchically clone the intact 106kb salinomycin gene cluster. The reliability of the cloning method has been proved by subsequently successful heterologous expression in *S. coelicolor* A3(2). Our results represent a potent approach to mine the function of the individual genes and identify novel and potentially useful analogues of the complex natural products through module exchange in the recipient.

## Methods

### Strains, plasmids and culture conditions

The bacterial strains and plasmids used in this study are shown in Table S1. All primers were synthesized by Sigma-Genosys (Germany) (Table S2). All restriction enzymes, Taq polymerase, and DNA markers were purchased from New England Biolabs (UK).

*E. coli* cells were cultured in Luria-Bertani (LB) liquid media or on LB agar (1.2% agar). Ampicillin (amp, 100 μg mL^−1^), kanamycin (km, 15 μg mL^−1^), chloramphenicol (cm, 15 μg mL), hygromycin (hyg, 30 μg mL^−1^), apramycin (apr, 15 μg mL^−1^), and tetracycline (tet, 5 μg mL^−1^) were added to the media as required.

For sporulation and conjugation, *S. coelicolor* A3(2) was grown on mannitol salt (MS) agar plates for 10 days. If necessary for conjugation, apr (50 μg mL^−1^) and nalidixic acid (NA, 50 μg mL^−1^) were added.

*S. albus* DSM41398, *S. coelicolor* A3(2), and mutant strains were cultivated in M1 medium (10 g L^−1^ starch, 4 g L^−1^ yeast extract, 2 g L^−1^ peptone) at 30 °C with constant agitation at 180 rpm.

### Bacterial genomic DNA isolation

*S. albus* DSM41398 was cultured in 30 mL medium (4 g L^−1^ glucose, 4 g L^−1^ yeast extract, 10 g L^−1^ malt extract, pH 7.2) at 30 °C for two days. After centrifugation, the cells were resuspended in 5 mL SET buffer (75 mM NaCl, 25 mM EDTA, 20 mM Tris, pH 7.5). After adding lysozyme to a final concentration of 1 mg mL^−1^ and incubating at 37 °C for 0.5–1 h, 500 μL 10% SDS and 125 μL 20 mg mL^−1^ proteinase K were added, and the mixture was incubated at 55 °C with occasional inversion for 2 h until the solution became clear. The solution was combined with 2 mL 5 M NaCl and 8 mL phenol:chloroform:isoamyl alcohol (25:24:1) and incubated at room temperature for 0.5 h with frequent inversion. After centrifuging at 4500 × *g* for 15 min, the aqueous phase was transferred to a new tube using a blunt-ended pipette tip, and the DNA was precipitated by adding one volume of isopropanol and gently inverting the tube. DNA was transferred to a microfuge tube, rinsed with 75% ethanol, dried under vacuum, and dissolved in ddH_2_O.

### Preparation of electrocompetent cells for recombineering

Recombineering and direct cloning were performed as described previously^7^ with several small modifications. The linear cloning vector p15A-amp, flanked with homology arms to target genes, was amplified by PCR using p15A-amp-ccdB^5^ as a template. Digested genomic DNA (10 μg) was mixed with 2 μg linear cloning vector and co-transformed into competent cells by electroporation.

### Conjugation

Conjugation between *E. coli* and *S. coelicolor* A3(2) was performed as described previously with minor modifications^45^. The plasmid containing the salinomycin gene cluster and elements for conjugation and integration was transformed into the donor strain *E. coli* ET12567 (pUZ8002). The donor strain was prepared by growth overnight at 37 °C in LB supplemented with antibiotics. The overnight culture was diluted 100-fold in 15 mL fresh LB plus antibiotic and grown at 30 °C to an OD_600_ of 0.3. The *E. coli* cells were washed twice with an equal volume of LB and resuspended in 0.1 volume LB. *S. coelicolor* A3(2) mycelia fragments were harvested from a four-day-old culture in TSB medium and served as the recipient strain. The donor strain and recipient strain were mixed with equal volumes, the mixture was centrifuged, and the supernatant was discarded. Finally, the pellet was resuspended in the residual liquid. The mating mixture was spread on MS plates and incubated at 30 °C. After 24 h, the cells were collected and spread on MS plates with NA (50 μg mL^−1^) and apr (50 μg mL^−1^) and further incubated at 30 °C until exconjugant colonies appeared.

### Extraction and analysis of the compound

*S. coelicolor*::*sal* gene cluster cells were cultivated in 300-mL flasks containing 30 mL M1 medium supplemented with apr (25 μg mL^−1^). The culture was grown at 30 °C with constant agitation at 180 rpm. After 13 days, the biomass was harvested by centrifugation, and 2% resin Amberlite XAD-16 was added to the supernatant before the resin was extracted with methanol. The received extracts were evaporated and dissolved in methanol and used for HPLC-MS analysis. The HPLC-MS measurement was performed on a Dionex Ultimate 3000 LC system utilizing a Waters Acquity BEHC-18 column (50 × 2 mm, 1.7-μm particle size). Separation of 2 μL sample was obtained using a linear gradient of A (water and 0.1% formic acid) and B (acetonitrile and 0.1% formic acid) at a flow rate of 600 μL min^−1^ at 45 °C. The gradient was initiated by a 0.5-min isocratic step at 5% B followed by an increase to 95% B over 9 min and a final 1.5-min step at 95% B before reequilibration with initial conditions. UV spectra were recorded by a DAD from 200–600 nm. MS measurement was carried on an amaZon speed mass spectrometer (Bruker Daltonics, Bremen, Germany) using the standard ESI source. Mass spectra were acquired in centroid mode ranging from 200–2000 *m/z* in positive ionization mode with auto MS^2^ fragmentation.

## Additional Information

**How to cite this article**: Yin, J. *et al.* Direct cloning and heterologous expression of the salinomycin biosynthetic gene cluster from *Streptomyces albus* DSM41398 in *Streptomyces coelicolor* A3(2). *Sci. Rep.* **5**, 15081; doi: 10.1038/srep15081 (2015).

## Supplementary Material

Supplementary Information
