# Supplementary material for: Direct cloning and heterologous expression of the salinomycin biosynthetic gene cluster from Streptomyces albus DSM41398 in Streptomyces coelicolor A3(2)
Source: Sci Rep. 2015 Oct 13;5:15081. doi: 10.1038/srep15081 (PMC4602208; doi:10.1038/srep15081)
Supplement: Supplementary Information [file srep15081-s1.pdf]

Supplemental Information

Direct cloning and heterologous expression of the salinomycin biosynthetic gene cluster from *Streptomyces albus* DSM41398 in *S. coelicolor* A3(2)

Jia Yin, Michael Hoffmann, Xiaoying Bian, Qiang Tu, Fu Yan, Liqiu Xia, Xuezhi Ding, A. Francis Stewart, Rolf Müller, Jun Fu and Youming Zhang

Table S1 Strains and plasmids

Strain or plasmid	Characteristics	References or sources
E. coli		
GB05	F-*mcr*A Δ(*mrr-hsd*RMS-*mcr*BC) *φ*80*lac*ZΔM15 Δ*lac*X74 *rec*A1 *end*A1 *ara*D139 Δ(*ara, leu*)7697 *gal*U *gal*K λ *rpsL* *nup*G*fhu*A::IS2 *rec*ET *redα*, phage T1-resistent	[1]
GB06	*end*A1*gln*V44 thi-1 *rel*A1 *gyr*A96 *rec*A1 *mcr*B+ Δ(*lac-pro*AB) e14- [F' traD36 proAB+ lacIq *lac*ZΔM15] *hsd*R17(rK-mK+) *rpsLrec*ET phage T1-resistent	
GB05-dir	GB2005, *ara*C-BAD-ETγA	[2]
GB05-red	GB2005, *ara*C-BAD-γβαA	[1]
GB05dir-gyrA462	GB05dir, *gyr*A462 (*ccd*B resistant)	[3]
GB05red-gyrA462	GB05red, *gyr*A462 (*ccd*B resistant)	[3]
ET12567(pUZ8002)	*rec*F, *dam-*, *dcm-*, CmR, KmR	[4]
Streptomyces		
S. albus DSM41398	Salinomycin-producing wild-type strain	DSMZ
S. coelicolor A3(2)	Recipient strain of gene cluster	[5]
S. coelicolor::sal	Salinomycin gene cluster in the chromosome, aprR	This study
Plasmid		
pSC101-BAD-Cre-tet	Cre under BAD promoter, tetR	[6]
pSC101-rha-ETgA-tet	RecET, redγ and RecA under rha promoter, tetR	This study
pBeloBAC11-amp-ccdB-4Sal	pBeloBAC11, the vector for direct cloing, ampR	This study
p15A-amp-F1	p15A replicon, contained the fragment of salO-salAIV, ampR	This study
p15A-amp-F2-lox71-neo-lox66	p15A replicon, contained the fragment of salAIV-salAVIII and the cassette of lox71-neo-lox66, ampR, kmR	This study
p15A-amp-F3	p15A replicon, contained the fragment of salAIX-orf18, ampR	This study

p15A-amp-F2	p15A replicon, the neo selection marker was deleted by Cre from the p15A-amp-F2-lox71-neo-lox66, ampR	This study
p15A-hyg-ccdB-F3-lox71-neo-lox66	p15A replicon, two cassettes of hyg-ccdB and lox71-neo-lox66 was integrated into the p15A-amp-salAIX-orf18, hygR, kmR	This study
p15A-amp-F2&3-lox71-neo-lox66	p15A replicon, contained the fragment of salAIV-orf18 and the cassette of lox71-neo-lox66, ampR, kmR	This study
pBeloBAC11-sal-lox71-neo-lox66	pBeloBAC11, contained the whole salinomycin PKS and the cassette of lox71-neo-lox66, kmR	This study
pBeloBAC-sal-int-attP-oriT-apr	pBeloBAC11, contained the whole salinomycin PKS, an origin of transfer(oriT), attP sequence and integrase gene, aprR	This study

Table S2. Oligonucleotides

Gene	Primer name	Primer sequence 5’-3’	Restriction enzymes sites	application
*sal*O	salO-5	AGTGAATTGTAATACGACTCACTATAGGGCGAATTCGAGCTCGGTACCCGGTGGACGAGACCCGACCGCG		BAC vector
	salO-3	GGCCCGCCAGCGGTCCATCA		BAC vector
*orf*18	Orf18-5	CTAATGAGCGGGCTTTTTTTTGAACAAAACAACTTATATCGTATGGGGCT**GGATCC**ttATGACCCGCCCCGCCCCCTG	*BamH*I	BAC vector
	Orf18-3	GTGACACTATAGAATACTCAAGCTTGCATGCCTGCAGGTCGACTCTAGAGaTCACCACGGCCACCCCTCGG		BAC vector
Amp-*ccd*B	Amp-ccdB-5	ATGCAGAGCGAACTGGCCACCCTGATGGACCGCTGGCGGGCCCGCCGCGCCGGGAGTTGA**GGATCC**TTTGTTTATTTTTCTAAATA	*BamH*I	BAC vector
	Amp-ccdB-3	AGCCCCATACGATATAAGTT		BAC vector
*sal*O-*sal*AIV	salO-salAIV-3	CGCTCGCGCCGGGAGCCGGGTTCGCGCTCCCGGCCGACCATGTGGATCTGCGAGAGGTAGCGGACCGCGCCG**GATATC**TTACCAATGCTTAATCAGTG	*Eco*RV	Direct cloning
	salO-salAIV-5	CCAGCTGGAGGCGGACCTGCTGTCCGTCGCCCTGGACAAGGACGAACGCAAGAACCTCACCCGGCGCCTCGAAG**GATATC**ACAACTTATATCGTATGGGG	*Eco*RV	
*sal*AIV-*sal*AVIII	salAIV-salAVIII-3	TCCGAGAGCAGCGGGGCGGGTCGCGCGGAGGTGAACAGCGGCCAGAACCGCTCCCAGTCCACGTCCGCGACGGCG**ATTAAT**ACAACTTATATCGTATGGGG	*Mse*I	Direct cloning
	salAIV-salAVIII-5	CGACGCGCTCCTCGAGCTCGGCGCCGCCCCGGTCGGCGAGACGGCCGCGCAGCAACCGCAGCAGGCCGCG**GCGATCGC**ACTGTA**GATATC**TTACCAATGCTTAATCAGTG	*Asi*SI&*Eco*RV	
*sal*AIX-*orf*18	salAIX-orf18-3	TCCGCTCCACGGGCGCGTCAGGAGTTGCTGTTGTCGAGGGCCAGCGAGACGAGGGCGTCGACGTCCATCTCG**GCGATCGC**ACAACTTATATCGTATGGGG	*Asi*SI	Direct cloning
	salAIX-orf18-5	TCGAGGACGACTTCGGCCTCGCCGTCGACCCCCGCCTCGCCCGCGAACTCCCCACCGTGACCGCCCTCGCCG**GATATC**TTACCAATGCTTAATCAGTG	*Eco*RV	
lox71-*neo*-lox66	sal-km-5	CCTGCTCGACGACGAACTCGAAACGAAATAACCCATGAACGGCGCCGCTTTACCGTTCGTATAATGTATGCTATACGAAGTTATTCAGAAGAACTCGTCAAGAAG		Direct cloning
	sal-km-3	AACTCAGTCACCTACCGGAAACAGGACCGGGCACGGGAAGCGGAAGGCCGTACCGTTCGTATAGCATACATTATACGAAGTTATGCTTGCAGTGGGCTTACAT		
Hyg-*ccd*B	hygccdb-5	TTACCAATGCTTAATCAGTGAGGCACCTATCTCAGCGATCTGTCTATTTCCCTAGGTTATATTCCCCAGAACATCA		Assembling
	hygccdb-3	ATGTATCCGCTCATGAGACAATAACCCTGATAAATGCTTCAATAATATTGAGGAGGCCTAGGTATGAAAAAGCCTGAACTCAC		
lox71-*neo*-lox66	kan-5	GTAGAGCGCCACCATCAGATGCGCGTGGAACTCCTCGCGCAGCGGGAACTTACCGTTCGTATAGCATACATTATAC		Assembling

		GAAGTTATGCTTGCAGTGGGCTTACAT	
	kan-3	GACGAGGAGGATGTTCTCGGCGGGCACCTCGAACTCCTCGGCGATCAGCATACCGTTCGTATAATGTATGCTATAC GAAGTTATTCAGAAGAACTCGTCAAGAAG	
Integrase-attP-oriT-Apramycin	intApSal5	TAGAGCGCCACCATCAGATGCGCGTGGAACTCCTCGCGCAGCGGGAACTTAGATCAGGCTTCCCGGGTGTC	Engineering
	intApSal3	CGCGGGCCGTGTCCCTCAAGTCCAGCAGGTCCGCGCGCAGTTGGCGGGTCTGACGCTCAGTGGAACG	
	attB-L	CAGGTTCACCCACAGCTG	Verification
	attB-R	CTCAACTAAAGTGGGGCG	Verification
	attP-dn	AAATGCCCGACGAACCTGAA	Verification
	attP-up	TCGCTATAATGACCCCGAAGCAG	Verification

Underlined sequences indicate homology arms.
Bold letters represent restriction enzyme sites.

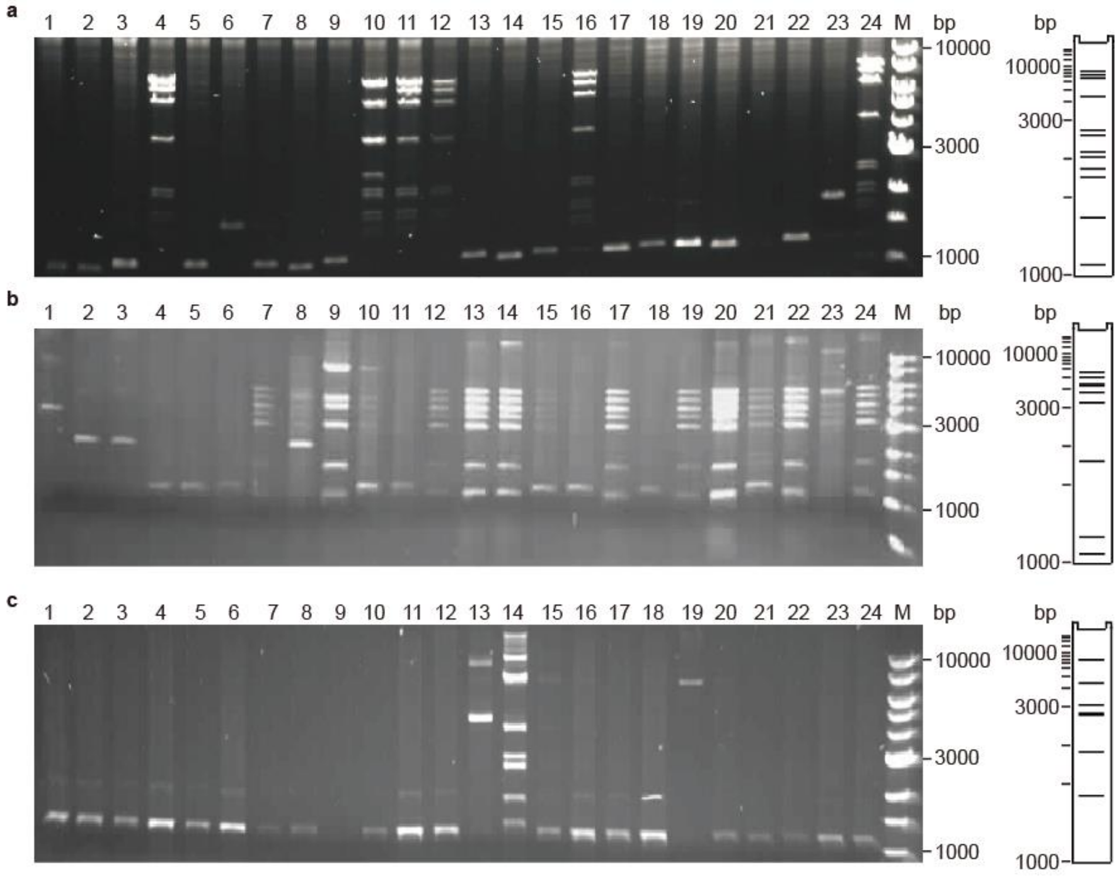

Figure S1 Digestion of the direct cloning products. **(a)** Products of fragment F1 were digested by *Nco*I. M, 1-kb NEB ladder. 1–24, clones obtained from direct cloning. **(b)** Products of fragments F2 and **(c)** F3 digested by *Pvu*II. M, 1-kb NEB ladder. 1–24, clones obtained from direct cloning.

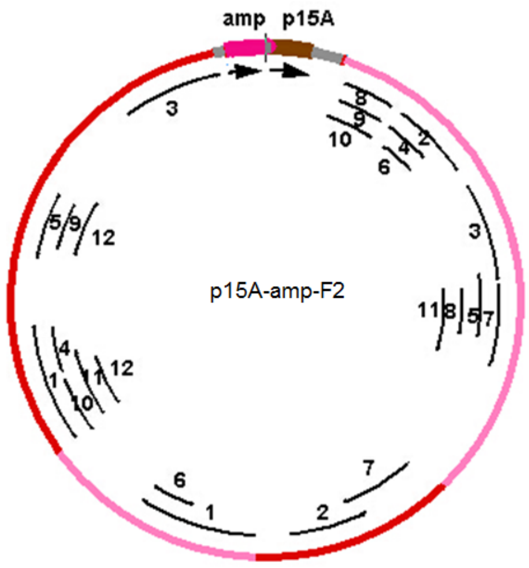

Figure S2 Alignment analysis of the F2 fragment and itself. The repetitive sequences were marked as same numbers.

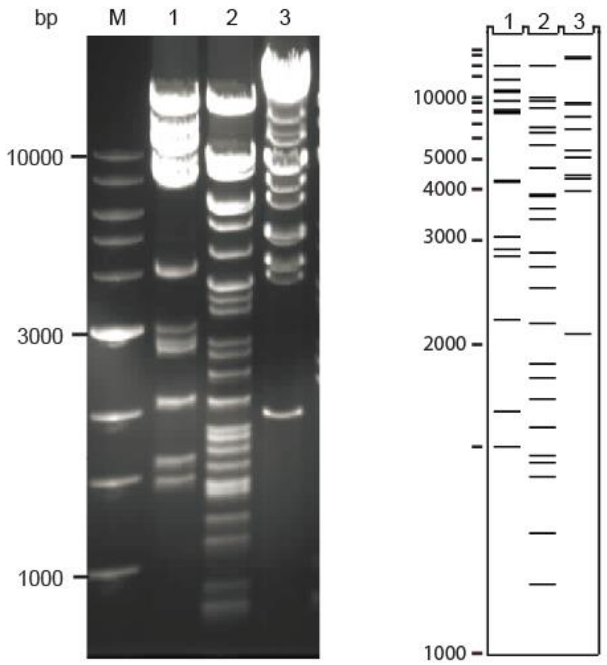

Figure S3 Digestion of pBeloBAC11-sal-lox71-neo-lox66. The three fragments were stitched into pBeloBAC11, and the correct clone was confirmed by three restriction enzymes: *Asc*I (1), *ApaL*I (2), and (*BgI*II (3). M: Marker (NEB 1-kb DNA ladder).

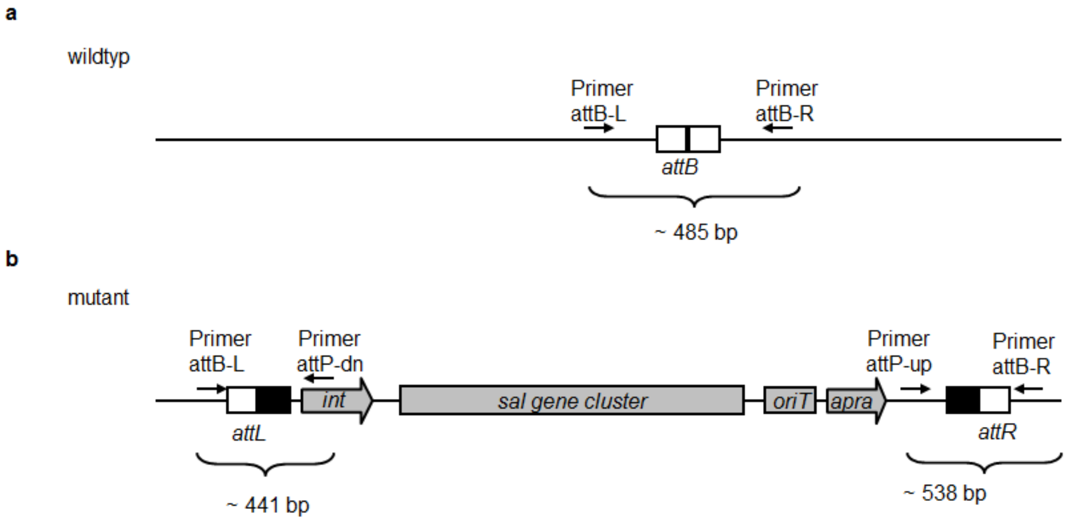

c

PCR No.	A	B	C
oligos	attBL/attB-R	attBL/attP-dn	attB-R/attP-up
wildtype	485bp	-	-
mutant	-	441bp	538bp

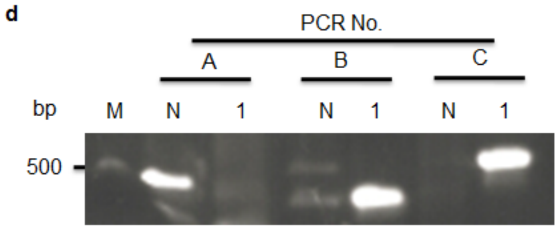

Figure S4 PCR verification of salinomycin gene cluster integration into the *att*B site in *S. coelicolor* A3(2). (**a, b**) Diagram of the PCR procedure in the wild-type (**a**) and mutant (b) strains using the indicated primers. Primers attB-L and attB-R were dependent on the sequence of *S. coelicolor* A3(2). Primers attP-down and attP-up were based on the BAC sequence. (**c**) PCR products and primer pairs. (**d**) Agarose gel containing PCR-amplified DNA fragments using to the primer pairs in (**c**). M, Marker; N, wild-type *S. coelicolor* A3(2); 1, exconjugant colonies.

References

1. Fu, J., Teucher, M., Anastassiadis, K., Skarnes, W. & Stewart, A.F. A Recombineering Pipeline to Make Conditional Targeting Constructs, Vol. 477. (Academic Press, Unit State; 2010).
2. Fu, J. *et al.* Full-length RecE enhances linear-linear homologous recombination and facilitates direct cloning for bioprospecting. *Nat. Biotechnol.* **30**, 440-446 (2012).
3. Wang, H. *et al.* Improved seamless mutagenesis by recombineering using ccdB for counterselection. *Nucleic Acids Res.* **42**, e37 (2014).
4. Paget, M.S.B., Chamberlin, L., Atrih, A., Foster, S.J. & Buttner, M.J. Evidence that the extracytoplasmic function sigma factor sigmaE is required for normal cell wall structure in *Streptomyces coelicolor* A3(2). *J. Bacteriol.* **181**, 204-211 (1999).
5. Huo, L., Rachid, S., Stadler, M., Wenzel, S.C. & Muller, R. Synthetic biotechnology to study and engineer ribosomal bottromycin biosynthesis. *Chem. Biol.* **19**, 1278-1287 (2012).
6. Anastassiadis, K. *et al.* Dre recombinase, like Cre, is a highly efficient site-specific recombinase in *E. coli*, mammalian cells and mice. *Dis. Model. Mech.* **2**, 508-515 (2009).
